# Detection of Microplastics and Heavy Metals Using Electronic Tongues and Machine Learning

**DOI:** 10.3390/s26103054

**Published:** 2026-05-12

**Authors:** Luis Angel Peña, Juan P. Hoyos-Sanchez, Juan Daniel Sarmiento, Mónica V. Sandoval Rincón, Diego A. Tibaduiza

**Affiliations:** 1 Dirección Académica, Universidad Nacional de Colombia, Sede de La Paz, La Paz 202017, Colombia; lpenad@unal.edu.co (L.A.P.); jhoyoss@unal.edu.co (J.P.H.-S.); mosandovalr@unal.edu.co (M.V.S.R.); 2 Department of Electrical and Electronics Engineering, Universidad Nacional de Colombia, Bogotá 111321, Colombia; jusarmientoa@unal.edu.co

**Keywords:** microplastics, electronic tongues, heavy metals, AAS, EPS, machine learning

## Abstract

Water resources face a significant environmental challenge: pollution from microplastics (MP) and heavy metals (HM). These elements pose a dual threat to ecosystems and public health. Microplastics, defined as particles smaller than 5 mm, are of anthropogenic origin, resulting from the degradation of plastics by environmental factors such as solar radiation and friction with the surrounding environment, as well as from their addition to cosmetic and textile products. These materials have been widely detected in drinking water and everyday foods. Heavy metals, high-density elements $\left(>5\;{g/\mathrm{cm}}^{3}\right)$, while naturally present in the Earth’s crust, are also generated in large quantities through human activity. Their toxicological risk lies in their ability to accumulate and efficiently move through the trophic chain. Due to the risks to public health and the impacts these pose to ecosystems, it is necessary to continue seeking solutions that enable their monitoring and detection. As a contribution, this work presents a methodology for detecting microplastics and heavy metals in seawater using different machine learning models and an electronic tongue coupled to a sensor network. Two different types of heavy metals, primarily zinc (Zn) and cadmium (Cd), as well as microplastic particles composed of expanded polystyrene (EPS), were detected under controlled conditions simulating different types of water. Atomic absorption spectroscopy (AAS) confirmed the concentrations of the heavy metals studied, supporting machine-learning classification of contaminated waters. Microplastics exhibited strong metal adsorption, influenced by the physicochemical properties of the water. Overall, AUC values above 90% were obtained for seven different models, demonstrating the reliability of the electronic tongue in conjunction with classical machine learning techniques for detecting these elements.

## 1. Introduction

The presence of microplastics and heavy metals in water represents one of the main environmental challenges facing humanity, due to the potential impacts on human [1,2] health and ecosystems [3]. Microplastics (MP) are particles smaller than 5 mm and have been detected in bodies of water used for human [4] consumption and in some commonly consumed foods such as salt [5]. Their origin is anthropogenic and is due to the fragmentation of this material through interaction with its surrounding environment [6]. They are also used in the cosmetics industry [7] as a raw material or obtained as a byproduct of different industries such as textiles [8] or the manufacture of polymer-based packaging. Heavy metals are metallic or metalloid elements characterized by relatively high density. They originate from both natural processes and anthropogenic activities, including mining, industrial operations, and technological applications [9,10]. Depending on their concentration, some heavy metals can exert toxic effects, including carcinogenicity, neurological damage, and disruption of biological systems [11,12]. They can also be classified into two categories: essential heavy metals and non-essential heavy metals. Essential heavy metals are those that living organisms need to carry out fundamental processes such as growth, metabolism, and the development of different organs; Non-essential heavy metals lack biological function and are toxic at low concentrations.

Despite their adverse effects, plastics have had a significant impact on humanity’s technological development; therefore, it is important to develop strategies to mitigate their short- and long-term effects. In this regard, the detection of these materials in different environments is of paramount importance worldwide. Historically, microplastics began to be detected in bodies of water in various studies around the world in the 1970s [13], although more recent research dates them back to the 1980s, with a significant turning point in 1998 when high levels of microplastics began to be detected in the North China Sea [14]. The most effective methods for detecting these contaminants are relatively slow and require expert analysis to implement and interpret the results. Puthongkham et al. [15] showed in a review that the use of machine learning and chemometrics for electrochemical sensors can be useful for detecting differences and classification tasks, in the same way in a previous work, detection and classification of microplastics using machine leraning was demonstrated by using impedance spectroscopy in PET particles [16]. From this point of view, electronic tongues are an interesting system for monitoring and automation processes such as the identification of heavy metals and microplastics. Therefore, the objective of this work is to develop agile methodologies that allow their identification in reasonably short timeframes. Currently, various studies have focused on the detection of these contaminants, including impedance spectroscopy. This technique has proven to be effective in identifying the major microplastics in freshwater environments [17,18]. Pulse voltammetry also shows significant progress in the detection of heavy metals [19,20]; however, these techniques have not been standardized within a common framework that would allow their use for the detection of any type of microplastic or heavy metal. This topic will be explored in greater depth in the following section, which explains in detail the most modern methods for detecting microplastics and heavy metals.

Recent work has demonstrated that electronic tongues can be applied to detect heavy Metal/Toxins, Shimizu et al. [21] work with a multisensory array based on non-specific sensors combined with multivariate statistical techniques as a solution for detecting emerging pollutants such as heavy metals and toxins in water. Gheibi et al. [22] developed an Expert System (ES) for automated removal of $Pb^{2+}$, $Co^{2+}$, and $Mn^{2+}$ using waste-derived concrete adsorbents, this system uses Genetic Algorithm (GA), Artificial Neural Networks (ANN), and Monte Carlo (MC) analysis. Putri et al. [23] introduced a machine learning approach to estimate microplastic concentrations in water using feature extraction of current-voltage signals from a potentiostat, ensemble learning, random forest, and SVM. A label-free method to differentiate clean microplastics from those contaminated with lead ions was proposed by [24], the method includes the use of electrochemical impedance spectroscopy (EIS) on screen-printed sensors and voltammetric analysis. In contrast to the revised work, this work presents the development of a methodology based on electronic tongues, sensor fusion, and artificial intelligence models as a technically and economically viable alternative for the real-time detection of microplastics and heavy metals, focusing on the detection of expanded polystyrene (EPS) and heavy metals cadmium (Cd) and zinc (Zn) in a saltwater environment.

This methodology is presented as a common framework upon which strategies for the early detection of these contaminants in bodies of water can be standardized.

## 2. Basic Concepts

This section presents some basic concepts about methods for the detection of heavy metals and microplastics, electronic tongues, and the techniques to be used in the methodology for detecting microplastics and heavy metals.

### 2.1. Methods for the Detection of Microplastics

Although there are different techniques for detecting microplastics, most of them require specialized equipment and personnel in laboratory conditions, and require significant technical capital for their collection and analysis.

There are different approaches to identifying microplastics, starting with machine learning using hyperspectral images [25] to identify various types of particulate matter. The primary limitation of this technique is the limited information available on microplastics in databases, as well as the relatively high margin of error, since the MP can be obscured by surrounding matter, making its identification complex and necessitating filtering processes. The polarized light scattering technique for detecting microplastics [26] presents essential advantages, since it allows the implementation of equipment in the field, and additionally, by controlling the polarization of the light, different types of materials can be detected. More traditional projects such as fluorescence microscopy [27] present a certain level of bias because their results can be interpreted as organic elements. One of the most widely used methods for the detection of microplastics is Raman spectroscopy [28]. This technique has high precision, but it is a slow process, especially when trying to identify the type of material. Additionally, it requires sample preparation, equipment calibration, and measurement by a specialist, which could increase its cost from a financial perspective. State-of-the-art techniques that propose the detection of microplastics using electronic tongues [29] and advanced electrochemical techniques, such as impedance spectroscopy [30,31], together with machine learning models, present a significant advance in the detection and classification by size of different types of microplastic particle [31]. In the following section, an analysis will be carried out in reference to the usefulness of electronic tongues in the detection of MP.

### 2.2. Methods for Heavy Metal Detection

Currently, several methods exist for detecting heavy metals. Below is a summary of some of the most popular techniques for heavy metal detection, focusing on their advantages and disadvantages.

1. Atomic absorption spectroscopy (AAS)

Atomic absorption spectroscopy (AAS) is one of the most widely used techniques for detecting heavy metals. This method is based on measuring the absorption of light by free metal ions in the gaseous state, enabling the identification and quantification of elements present in a sample. Its main advantages include high sensitivity, as it can detect concentrations at parts-per-million (ppm) and even parts-per-billion (ppb) levels. Furthermore, it is particularly effective for the analysis of metals such as lead, cadmium, mercury, and arsenic in matrices including water, soil, and biological samples [32]. However, this technique also presents several limitations. It generally requires prior sample preparation, which can be complex and time-consuming. Additionally, it is typically limited to the analysis of one element at a time, reducing its efficiency in multielement studies. It also involves the use of specialized instrumentation and relatively high costs, as well as the need for trained personnel for proper operation and interpretation of the results.

2. Inductively coupled plasma mass spectrometry (ICP-MS)

ICP-MS is an advanced analytical technique that combines high-temperature plasma with mass spectrometry. It offers exceptional sensitivity, capable of detecting metals at parts per billion (ppt) levels [33].

Furthermore, it allows the simultaneous analysis of multiple elements, making it widely used in environmental monitoring, clinical diagnostics, and food safety control. Among its advantages are its high precision, multi-element capability, and very low detection limits. However, it has significant disadvantages, such as the high cost of acquiring and maintaining the equipment, the need for specialized infrastructure, and the dependence on highly trained personnel. Additionally, its availability may be limited in contexts with financial constraints or in laboratories with less technical capacity.

3. X-ray fluorescence spectroscopy (XRF)

XRF is a non-destructive technique that identifies and quantifies heavy metals by measuring the characteristic X-rays emitted by a sample when excited with high-energy radiation. It requires minimal sample preparation and can be implemented on portable equipment, making it ideal for field analysis and rapid assessments.

Its main advantages are speed, portability, low sample preparation requirements, and the possibility of in situ analysis [34]. However, its sensitivity is lower compared to techniques such as ICP-MS, especially for very low concentrations. Furthermore, it can have limitations with complex matrices, and although portable equipment exists, the most accurate devices can be expensive.

4. Electrochemical Techniques (Voltametry and Potentiometry)

Electrochemical techniques detect heavy metals from the electrical signals generated during chemical reactions. They are cost-effective, portable methods suitable for on-site analysis. Within this group, electronic tongue systems, which combine sensor arrays with pattern recognition techniques, represent an emerging approach for the simultaneous detection of multiple contaminants.

Their advantages include low cost, ease of use, portability, and the possibility of integration with machine learning systems. However, they can be susceptible to interference, have lower selectivity in some cases, and require frequent calibration. Furthermore, the development and implementation of electronic tongues can demand specialized technical expertise.

5. Inductively Coupled Plasma Optical Emission Spectroscopy (ICP-OES)

ICP-OES is based on the analysis of light emitted by excited atoms and ions in a plasma. It offers multi-element capability and good sensitivity, generally in the parts-per-billion (ppb) range. It is widely used to analyze heavy metals in environmental, industrial, and agricultural samples.

Its advantages include the ability to analyze multiple elements simultaneously, good accuracy, and lower cost compared to ICP-MS. Disadvantages include lower sensitivity than ICP-MS, the need for specialized equipment, significant gas and energy consumption, and the requirement for trained technical personnel to operate it.

6. Colorimetric and Spectrophotometric Methods

These traditional methods are based on chemical reactions that generate colored compounds, the intensity of which is measured by spectrophotometry [35]. They remain useful for routine monitoring due to their simplicity and low cost. Their advantages include accessibility, low cost, ease of implementation, and reduced need for highly specialized personnel. However, they have limitations in sensitivity and selectivity, especially in the presence of interfering substances, and are generally less precise for trace analysis compared to advanced instrumental techniques.

7. Biosensors

Biosensors employ biological recognition elements, such as enzymes, antibodies, or microorganisms, coupled to transducers to detect heavy metals. They offer high specificity, rapid response, and miniaturization potential. Recent advances include genetically modified organisms capable of generating detectable signals in the presence of specific metals. Their advantages include high selectivity, rapid response, portability, and potential for real-time applications. However, they can present stability problems, a limited lifespan, sensitivity to environmental conditions, and, in some cases, require complex development processes and specialized personnel.

8. Neutron activation analysis (NAA)

NAA is a highly sensitive nuclear technique that involves irradiating samples with neutrons and measuring the resulting radioactive emissions. It allows the detection of trace amounts of heavy metals without destroying the sample, making it especially valuable for unique or irreplaceable materials [36].

Its advantages include high sensitivity, precision, and non-destructive nature. However, its disadvantages include very high costs, low availability, the need for specialized nuclear facilities, and strict safety requirements, as well as the need for highly trained personnel.

The choice of method for detecting heavy metals depends on several factors, such as the specific application, analysis time, number of analytes, required portability, and financial, technical, and equipment and personnel availability constraints. In this context, the integration of electrochemical techniques with machine learning is emerging as a promising alternative for developing more accessible, efficient, and adaptable systems for different environments.

### 2.3. Cross-Selectivity

Cross-selectivity is a key concept in electrochemical analysis, especially when seeking to detect a specific analyte in the presence of other compounds with similar electrochemical properties. In simple terms, it refers to a sensor’s ability to distinguish between the target analyte and potential interferents present in the sample. In electrochemical sensors, this phenomenon becomes relevant because many compounds can present similar redox signals or interfere with the measurement. For example, in glucose monitoring, substances such as ascorbic acid or uric acid can generate electrochemical responses that confound the result if the sensor is not sufficiently selective. Analyte characterization must consider this parameter to ensure measurement reliability. Assessing cross-selectivity involves subjecting the sensor to different conditions and potential interferents, measuring how its response varies with compounds other than the target analyte. To improve selectivity, sensors can be modified with specific materials, such as enzymes, conductive polymers, or functionalized electrodes, which enhance affinity or recognition of the analyte. In the case of screen-printed sensors, this translates into printed layers on the working electrode that improve chemical discrimination [37,38].

### 2.4. Electronic Tongues

Electronic tongues are sensor systems [39] designed to emulate the human sense of taste, capable of recognizing chemical patterns in liquid solutions. In the field of electrochemistry, these tongues are made up of arrays of partially selective electrochemical sensors that respond to various compounds, generating signals that are interpreted using multivariate analysis techniques such as principal component analysis (PCA) [40] or artificial intelligence models.

Some of the electrochemical techniques used in an electronic tongue are voltammetry [41], amperometry [42], potentiometry, and electrochemical impedance spectroscopy [43]. Each of these techniques records distinct signals—such as variations in current, potential, or impedance—in the presence of multiple analytes, giving these systems the ability to obtain unique “chemical fingerprints” for each sample.

One of the most notable applications of these technologies is in the environmental sector [44,45], where they can detect contaminants in water bodies, such as heavy metals, pesticide residues, or volatile organic compounds. Thanks to their high sensitivity and portability, electronic tongues can be used for real-time monitoring of water sources, treatment plants, or sensitive natural environments.

In the food industry [46], these systems have proven extremely useful for assessing the quality, authenticity, and freshness of products such as wines, oils, juices, and dairy products. They are also used to detect adulteration, control fermentation processes, or verify product stability during storage. The ability of electronic tongues to generate complex and reproducible flavor or chemical composition profiles makes them valuable tools for automated quality control and analysis in complex matrices, where traditional methods can be slow or insufficient.

### 2.5. E-Tongue and Machine Learning Models in Environmental Analysis

Electronic tongues are devices that use a network of sensors with a high margin of cross-selectivity. However, its initial focus was oriented towards the parameterization of food; it has since begun to be used in various fields with significant results [47]. The data structure delivered by an electronic tongue varies depending on the electrochemical technique used; likewise, the information is delivered in matrices considering the data from each sensor. The high levels of cross-selectivity imply that the data have a significant variation from one sensor to another, so a pre-processing and deployment stage is crucial that allows the creation of data classes with the same characteristics as well as standardizing and normalizing the information, to avoid bias in machine learning models.

**Figure 1:**
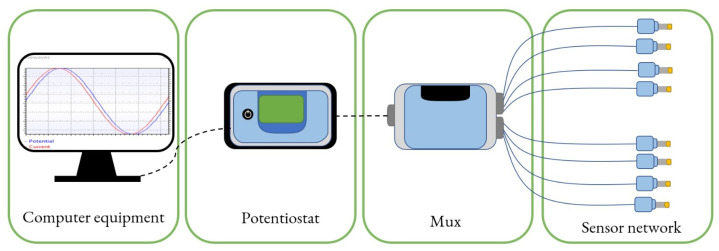
Four-stage electronic tongue architecture used in this work.

There are different electronic tongue architectures [48] in the industry. Their design depends on their field of application, such as pharmaceutical applications [49], beverage characterization [50], oil classification [51], food characterization [42,52], or sensory analysis [53]. Another essential aspect that determines the type of tongue is the sensors that make up its detector network, as well as the detection technique. Figure 1 shows the electronic tongue architecture used for the measurements in this work. The tongue’s data input is a network of electrochemical sensors screen-printed on ceramic, with different materials in their working electrodes, generating data with high cross-selectivity. The multiplexer (MUX) channels the excitation signals sent from the potentiostat to the sensors and receives the response from the network, one sensor at a time, sending the data back to the potentiostat. The potentiostat, in turn, is the instrument that generates the voltage signals necessary to stimulate the sensor network, receives the response from these stimuli, and sends them to the computer system for processing.

The machine learning models used in this work have several characteristics that allow them to address the complexity of environmental data. The Multilayer Perceptron (MLP), as a multilayer artificial neural network, stands out for its ability to model complex nonlinear relationships. It is widely used in the interpretation of signals from electrochemical sensors and spectroscopic data to estimate pollutant concentrations and classify microplastics; however, it requires large volumes of data and proper hyperparameter [54] fitting. The Support Vector Machine (SVM), on the other hand, is based on the construction of an optimal hyperplane that maximizes the separation between classes, making it especially useful in the classification of microplastics and in the identification of the presence of heavy metals in high-dimensional datasets, offering robustness against overfitting. Random Forest models utilize multiple decision trees to improve accuracy and reduce variance, enabling the handling of noisy data, the identification of relevant variables in chemical sensors, and the efficient classification of environmental samples. Complementarily, Gradient Boosting builds models sequentially, with each new model correcting the errors of the previous one, achieving high accuracy in predicting pollutant concentrations and detecting complex patterns in spectroscopic data. However, it can be computationally demanding and susceptible to overfitting if not properly regulated. Similarly, Bootstrap Aggregating contributes to improved model stability by generating multiple subsets of data, reducing variance and increasing robustness to noise present in experimental measurements, especially in sensor-based systems. Finally, the K-Nearest Neighbors (KNN) algorithm is based on data similarity, classifying new observations according to their proximity to their nearest neighbors in the feature space. This makes it useful for spectrum classification and pattern identification in environmental databases; however, its performance depends on the appropriate selection of the distance metric and the number of neighbors considered. Together, these models are presented as complementary tools that allow for the efficient detection and classification of heavy metals and microplastics in diverse environmental settings, such as seawater.

## 3. Methodology

**Figure 2:**
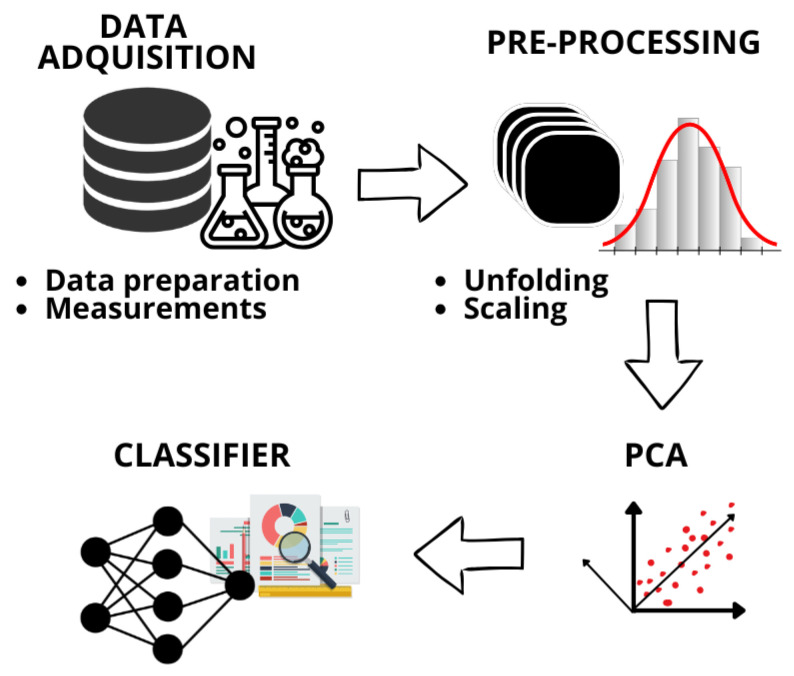
Illustration of the methodology proposed in this work.

The classification of marine water samples follows a series of steps, from data acquisition to final classification using visualization tools such as Principal Component Analysis (PCA) and algorithms for detection and classification. Figure 2 shows the steps considered in this work for the development of the methodology that allowed the detection and classification of microplastics and heavy metals.

The next subsections present more details on each step of the methodology and the particular way in which it is implemented in the laboratory.

### 3.1. Data Acquisition

In this work, an electronic tongue system, composed of seven sensors made of different materials, was connected to a multiplexer and a PalmSens4 potentiostat from the PalmSens company (Utrecht, The Netherlands) using PSTrace4 software for data collection. Table 1 shows the material used for each measuring electrode in the tongue sensor array.

### 3.2. Electrochemical Technique Used

The technique used to stimulate the working electrodes in the sensor network falls under the category of amperometric techniques. These techniques focus on increasing sensor sensitivity and measuring the chemical species that make up a solution. This approach is optimal for detecting heavy metals and particles, such as microplastics.

#### 3.2.1. Multistep Amperometry

**Figure 3:**
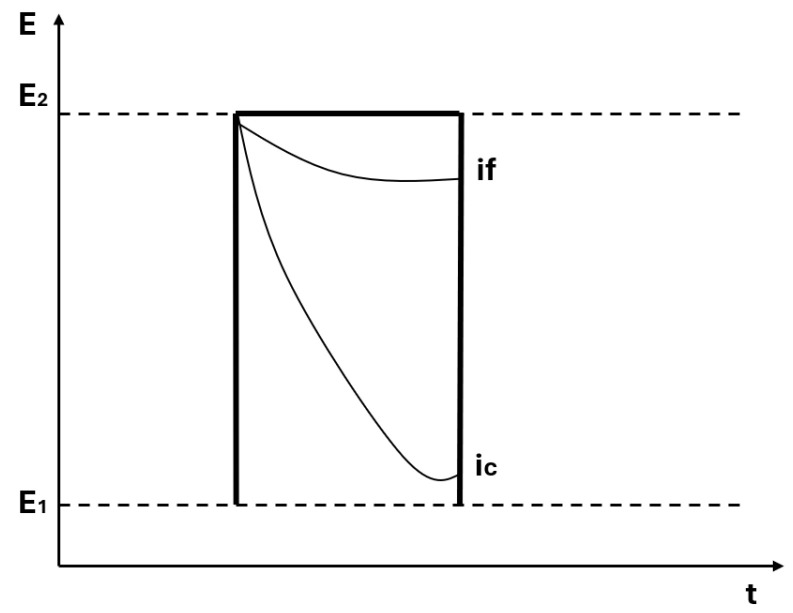
Type of signal applied when the technique of pulse voltammetry.

In multistep amperometry, the working electrode is stimulated with a pulsed voltage signal, as shown in Figure 3.

**Figure 4:**
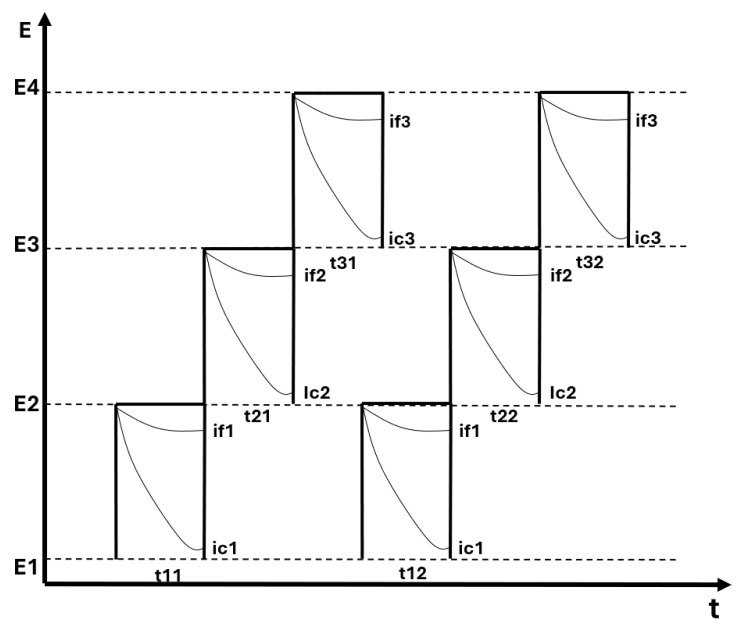
Applied potential in pulse voltammetry.

The objective in this inspection technique is to obtain a measure of the faradic currents (if) at each working electrode. In this way, the inspection considers several steps at different potential levels to obtain different current levels as in Figure 4.

For this work, a single-cycle voltage signal with 10 voltage levels (five positive and five negative) and a pulse window between −0.5 V and +0.5 V was applied. This type of signal and the number of cycles are applied by considering previous results [31]. To ensure that each of the potentials applied at the different steps remained constant and reproducible throughout the experiment, a constant pulse width of 0.1 s was configured for the reference electrode, guaranteeing the quality and stability of the current signal measured at each potential step. Similar configurations have been successfully validated in machine learning models for food classification [55].

**Figure 5:**
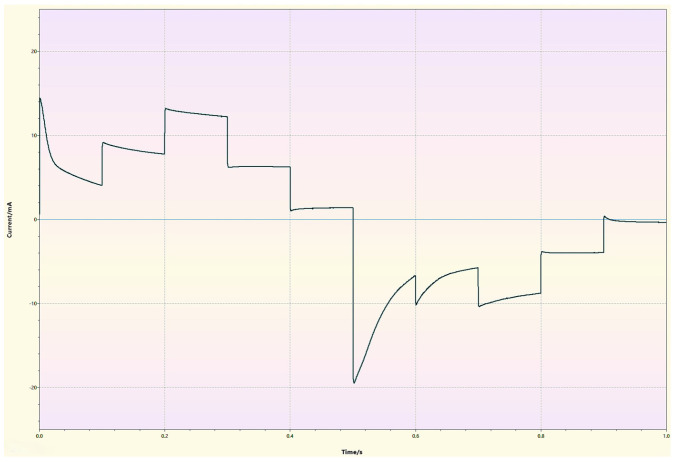
Response of a sensor with a platinum electrode in a synthetic seawater solution containing cadmium.

As an example of the type of signals from the sensors, Figure 5 shows the response of a sensor with a platinum working electrode in a sample of synthetic water solution containing cadmium.

#### 3.2.2. Samples

Table 2 shows the different types of samples that were used for the measurements, taking three of them as a base:deionized water (DW) with electrical conductivity 0.05 μS/cm, synthetic saltwater (SSW), and seawater (SW). The synthetic seawater (SSW) was prepared by dissolving analytical-grade reagents in deionized water. The reagents used were NaCl (Merck, CAS 7647-14-5, Emsure, ISO, Reag. Ph. Eur), MgCl_2_ · 6H_2_O (Merck, CAS 7786-30-3, Emsure, ISO, Reag. Ph. Eur), KCl (PanReac AppliChem, CAS 7447-40-7, Reag. USP for analysis, ACS, ISO), and CaCO_3_ (PanReac, CAS 471-34-1, USP, BP, Ph. Eur, pure, pharma grade). The prepared SSW contained 6.85 g/L NaCl, 2.68 g/L MgCl_2_, 0.19 g/L KCl, and 0.26 g/L CaCO_3_. The seawater samples were taken in the Caribbean Sea at a depth of 14 m, and at a distance of 12.7 km from the beach (geographic coordinates 11°05′51.411″ N, 74°20′10.5″ W) in Cienaga, Magdalena, Colombia. To minimize chemical or biological alterations, the seawater samples were acidified to approximately pH 2 (HNO_3_ Merck, CAS 7697-37-2) at the sampling point, and subsequently refrigerated, following the guidelines of NTC-ISO 5667-3 (Water quality-Sampling-Preservation and handling of water samples [56]). Artificial heavy metal contamination was performed using stock standard solutions of Cd (Panreac, code 313175.1208, 1000 mg/L for AAS) and Zn (Panreac, code 313193.1208, 1000 mg/L for AAS). Each sample was prepared with a final volume of 500 mL of water and an initial concentration of 1.0 mg/L of Zn and 1.5 mg/L of Cd. To each sample, 0.2 g of expanded polystyrene (EPS) microplastic, with a particle size of 250 μm, was added to achieve a microplastic concentration of 0.4 g/L. According to Table 2 and the availability of seawater samples, 23 classes of samples were analyzed.

**Figure 6:**
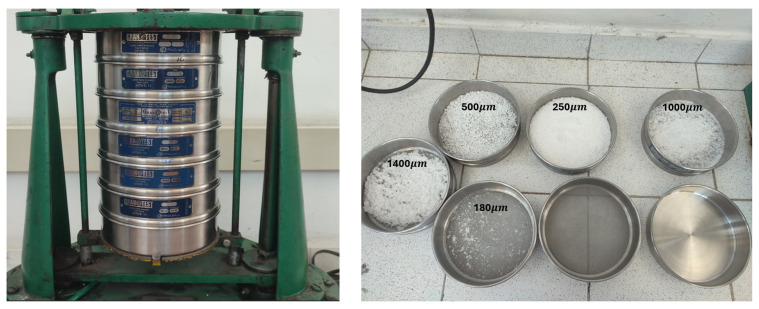
Microplastic particles separated by size.

The microplastic was obtained from segments of material processed using a shredding and cutting machine. These segments then underwent a sieving process to separate them into different sizes. The result of the sieving process is shown in Figure 6.

**Figure 7:**
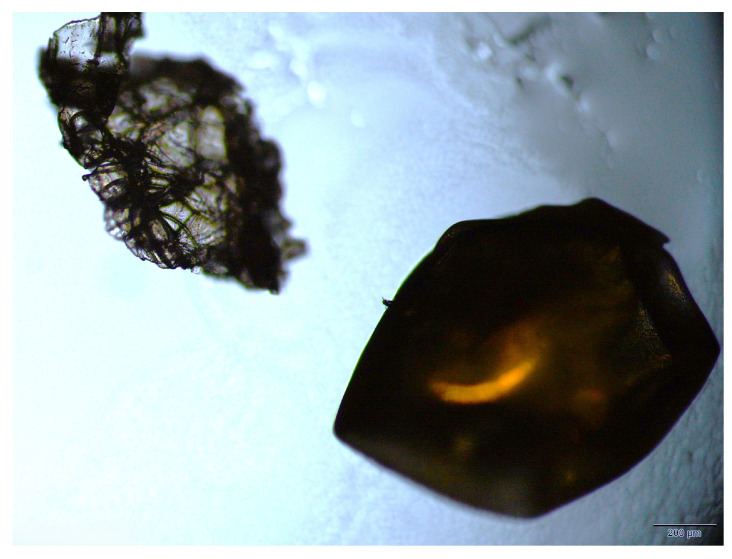
Expanded polystyrene microplastic particle next to a salmon egg. Magnification 2.52×.

Although the microplastic particles were separated by size, due to their asymmetrical morphology, a precise size was not obtained; however, the separation process allowed for a sufficiently good approximation to perform the measurements. The Figure 7 shows a sample with an ESP particle next to a salmon egg at a scale of 500 μm.

**Figure 8:**
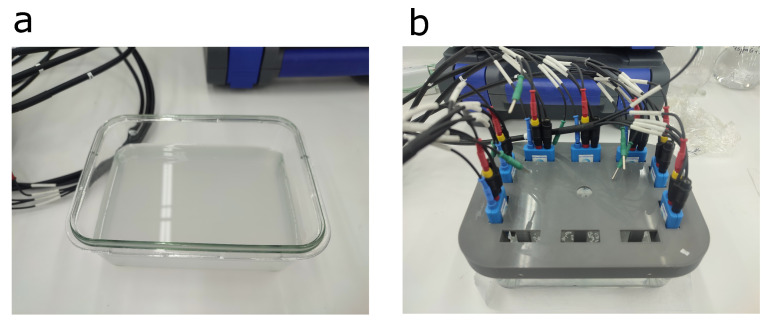
(**a**) Rectangular glass container for measurements. (**b**) lid with seven measurement electrodes.

Once prepared, each sample was placed in a rectangular glass container. For data acquisition, a specifically designed lid incorporating seven measurement electrodes was used (see Figure 8). Subsequently, a total of 200 measurements were conducted for each sample under consistent experimental conditions. Upon completion of the measurements for each sample type, both the container and the electrodes were rinsed with distilled water to prevent cross-contamination prior to the evaluation of the next sample.

Given the high salinity of the seawater matrix (0.5 M NaCl), potential issues such as electrode drift, fouling, and baseline shifts were carefully considered. To minimize electrode fouling and signal carryover effects, all electrodes were thoroughly rinsed with deionized water after each measurement, ensuring consistent surface conditions across samples. Additionally, signal normalization was applied prior to model training, which mitigates baseline variations associated with the high ionic strength of the medium. This preprocessing step ensures that the models focus on relative signal patterns rather than absolute signal magnitudes. Furthermore, the use of machine learning models such as Random Forest, Support Vector Machines, and Neural Networks enhances robustness against baseline shifts, as these models learn discriminative features based on the overall signal structure rather than relying on absolute current values.

### 3.3. Preprocessing

The pre-processing and organization of data are crucial for data analysis applications, including AI-based strategies [18]. Pre-processing involves cleaning, normalizing, and transforming the raw data into a format that is suitable for input into AI algorithms. This step helps improve the quality of the data and enhances the performance of AI models. On the other hand, organizing the data involves structuring it in a way that makes it easier for AI algorithms to learn and make predictions. Proper data organization can lead to more accurate results and better decision-making by AI systems. In this sense, this paper explores various strategies for pre-processing and data organization to enhance the results in identifying microplastics and heavy metals in seawater.

During data acquisition, *J* = 7 sensors (S) were used in a number of *I* = 200 measurements (M). For each measurement, the sensors individually resulted in a signal with a resolution of *K* = 2501 sample points (p). Given this, a data reorganization process was carried out using three alternative methods, which are proposed below.

#### 3.3.1. Data Unfolding

Let $X\;\in \;R^{I\times J\times K\times Q}$ denote the four-way data array, where *I* is the number of measurements, *J* is the number of sensors, *K* is the number of sample points per sensor signal (p) and *Q* is the number of classes. In order to apply standard two-dimensional machine learning algorithms, this three-way array must be rearranged into a two-dimensional matrix. Following the notation of Westerhuis et al. [57], the four-way array can be unfolded in multiple ways depending on which mode is preserved as the object direction. In this work, three unfolding strategies were evaluated and each method is explained below.

**Figure 9:**
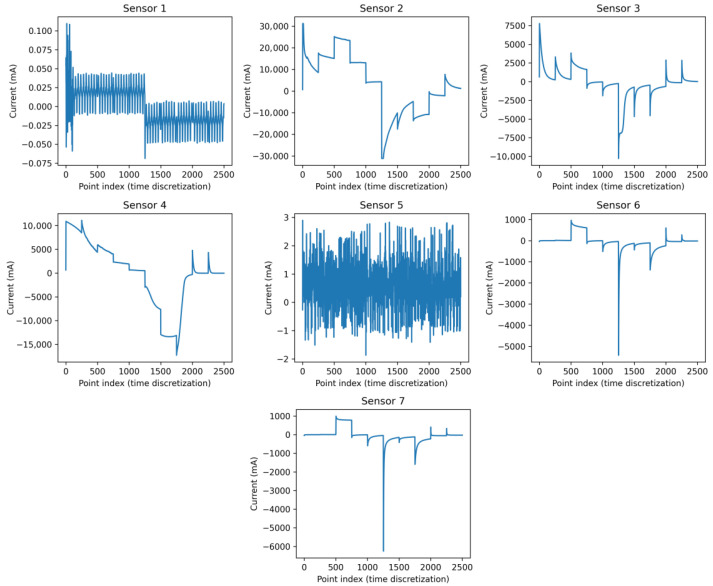
Graph of the volt-ampere response for each sensor during a measurement of a seawater sample containing cadmium, zinc, and microplastics. The graph shows the response in amperes for the discrete data points over a time interval.

**Figure 10:**
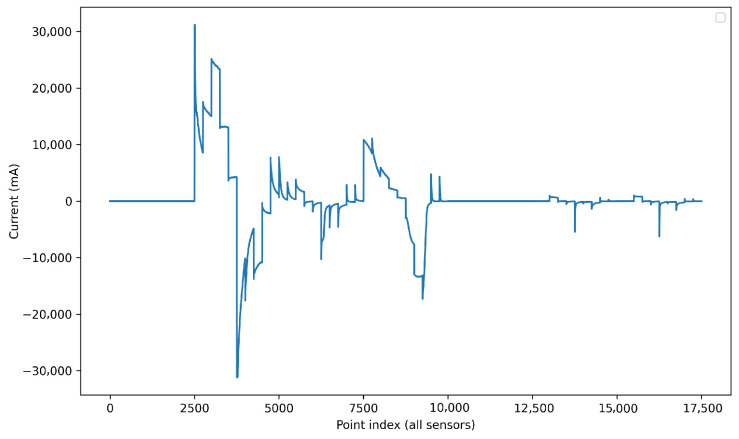
This graph results from the visualization of data for a seawater measurement of cadmium, zinc, and microplastics. Since it is generated by connecting the data points from the 7 sensors—each containing 2501 data points—the resulting graph consists of 17,507 data points, each representing a specific characteristic.

**Figure 11:**
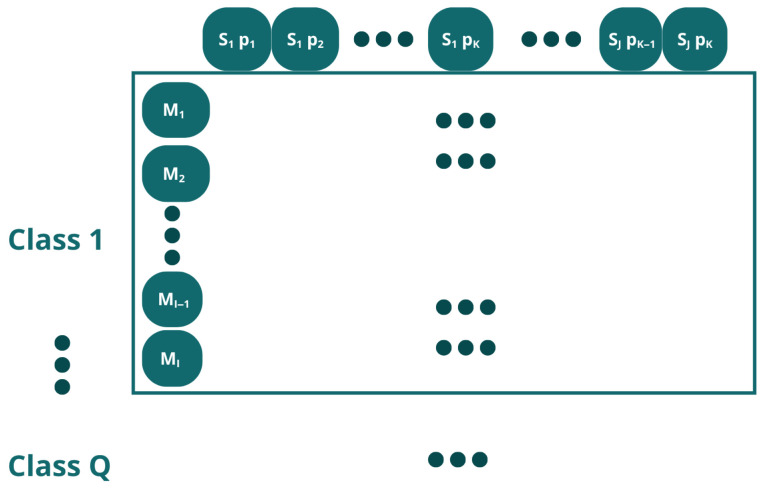
Data Organization Structure in the Unfolding Method 1. Each class has a number I of measurements (M), and the characteristics are determined by each point (p) on the graph generated by each sensor (S), with a total number of points generated per sensor K and a total number of sensors J.

**Figure 12:**
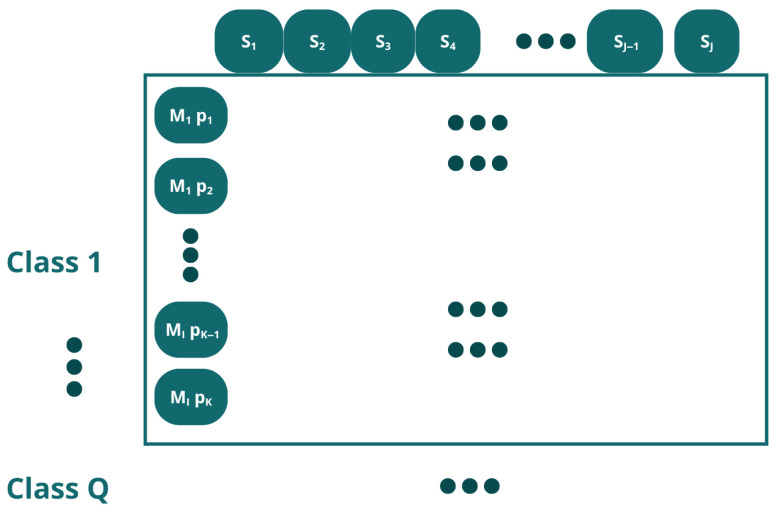
Structure of the data organization using unfolding method 2.

**Figure 13:**
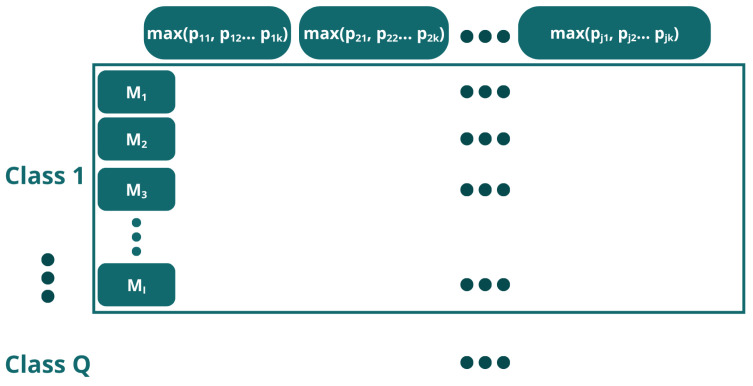
Data organization structure using unfolding method 3. Each class contains a number I of measurements (M), and the characteristics are determined by the maximum value recorded by each sensor.

- Method 1
  In Method 1, each data point represents a time-discretized point on the volt-ampere curve from a measurement taken by a sensor. Each measurement from each sensor comprises 2501 data points (Figure 9); since there are 7 sensors, the total number of data points is 7 × 2501 = 17,507 (Figure 10).
  This unfolding data method is shown in Figure 11. In this approach, the measurement direction is preserved so that each row corresponds to one complete measurement across all sensors and all time points. Then, a matrix of I × (J · K) for each class is defined, and the resulting matrix has dimensions (I · Q) × (J · K), where *Q* is the number of classes. Formally, for measurement *i* of class *q*, the row vector is constructed by concatenating the *K*-point signal of each of the *J* sensors:
  (1) $$x_{i}=\left[x_{i,1,1},\ldots ,x_{i,1,K},\;x_{i,2,1},\ldots ,x_{i,2,K},\;\ldots ,\;x_{i,J,1},\ldots ,x_{i,J,K}\right]\in R^{J\cdot K}$$
- Method 2
  In this approach, each individual time point of each measurement is treated as a separate observation, as shown in Figure 12. The resulting matrix has dimensions (I · K · Q) × J, where each row corresponds to one time instant of one measurement, and the *J* columns correspond to the simultaneous readings of the *J* sensors at that instant:
  (2) $$x_{i,k}=\left[x_{i,1,k},\;x_{i,2,k},\;\ldots ,\;x_{i,J,k}\right]\in R^{J}$$
  Although this strategy substantially increases the number of rows, it reduces the number of predictors to *J*, resulting in lower memory requirements per row at the cost of a much larger dataset.
- Method 3
  This method does not correspond to a standard unfolding of the three-way array. Instead, to reduce the characteristics while preserving the number of inputs, it applies a feature extraction step by retaining only the maximum value across the *K* sample points *p* for each sensor in each measurement:
  (3) $${\tilde{x}}_{i,j}=\max_{k\in \{1,\ldots ,K\}}x_{i,j,k}$$
  The resulting matrix has dimensions (I · Q) × J as shown in Figure 13, identical in shape to Method 2 but with far fewer rows. This approach achieves the maximum reduction in dimensionality while preserving the number of observations.

#### 3.3.2. Dataset

For the dataset construction process, only 10% of the samples were used in Methods 1 and 3, whereas Method 2 used only 0.1%, as summarized in Table 3. This reduction substantially decreased sampling time and ensured fast response times in practice. From the selected samples, 80% was used to construct the training dataset and the remaining 20% to form a held-out test set, which was reserved exclusively for final performance evaluation and never used during model selection or hyperparameter tuning. To control overfitting and improve generalization, 5-fold cross-validation with regularization was applied exclusively over the training subset during model fitting. All performance metrics reported in the section experimental results (AUC, accuracy, F1-score, among others) correspond to evaluation on the held-out test set.

#### 3.3.3. Scaling

Due to the scale difference caused by variations between sensors and samples, a data scaling process was performed to normalize the data. For this, Autoscaling or Z-score was used, which involves subtracting the mean μ calculated for each column of the data set from each value, and then dividing the result by the standard deviation σ also calculated for the values in each column

(4) $$z=\frac{x-\mu }{\sigma }$$

the equation adapted for each method is presented in the following

- Method 1:
  $$\mu_{jk}=\frac{\sum_{i}^{I}x_{ijk}}{I}\;\sigma_{jk}=\sqrt{\frac{\sum_{i}^{I}{\left(x_{ijk}-\mu_{jk}\right)}^{2}}{I}}$$
- Method 2:
  $$\mu_{j}=\frac{\sum_{i}^{IK}x_{ij}}{IK}\;\sigma_{j}=\sqrt{\frac{\sum_{i}^{IK}{\left(x_{ij}-\mu_{j}\right)}^{2}}{IK}}$$
- Method 3:
  $$\mu_{j}=\frac{\sum_{i}^{I}x_{ij}}{I}\;\sigma_{j}=\sqrt{\frac{\sum_{i}^{I}{\left(x_{ij}-\mu_{j}\right)}^{2}}{I}}$$

### 3.4. Dimensionality Reduction

For feature extraction and dimensionality reduction, the Principal Component Analysis (PCA) algorithm was used. The purpose of its use was to reduce the number of predictor variables from *N* to *P*, where P < N, thus allowing us to condense the data variability into a smaller set of variables and enable graphical representations in two or three dimensions. Previous works have demonstrated that plots of the first components are a good visualization tool when the retained variance in the first components is high [58]. This algorithm makes use of the covariance matrix. For a dataset with *n* observations, the covariance matrix is given by

$$\Sigma \left(X\right)=\frac{1}{n-1}X^{T}X$$

with dimensions n × n. By computing eigenvalues *v* and eigenvectors λ, the solution to the following equation is obtained:

Σv=λv

finally, sort the eigenvalues from largest to smallest and select the first *p* that would be the most important principal components [59].

### 3.5. Classification

The components obtained from PCA were used as input features to the classifiers. Seven classification algorithms were selected to represent a diverse range of learning strategies and decision boundary complexities. Ensemble methods were included due to their demonstrated robustness in high-dimensional classification tasks. Bagging-based algorithms such as Random Forest [60] and Bagging [61] aggregate predictions from multiple decision trees trained on random data subsets, reducing variance without increasing bias. Boosting-based methods such as Gradient Boosting [62] build models sequentially, where each estimator corrects the residual errors of its predecessor. Instance-based approaches such as K-Nearest Neighbors [63] classify new observations based on proximity in the feature space, requiring no assumptions about the underlying data distribution. Linear models such as Logistic Regression [64] applies regularization to prevent overfitting and provide interpretable decision boundaries for linearly separable problems, and in our paper, it is the baseline model. Support Vector Machines [65] seek the hyperplane that maximizes the margin between classes, offering strong generalization in high-dimensional spaces. Finally, the Multilayer Perceptron (MLP) [59] represents a feedforward neural network capable of capturing nonlinear relationships between the extracted features and the target classes.

To improve classification performance, hyperparameters were optimized using grid search. The search was conducted exclusively over the training subset to prevent data leakage into the held-out test set. Table 4 summarizes the hyperparameters and search ranges evaluated for each model. All experiments employed a five-fold cross-validation scheme with a fixed random seed to ensure reproducibility.

### 3.6. Evaluation

The evaluation of the models is performed by using several performance metrics. Among them, the area under the receiver operating characteristic curve (AUC-ROC) is highlighted, since it is one of the most widely used measures for assessing the discriminative ability of a classifier. The ROC curve plots the true positive rate (sensitivity) against the false positive rate (1 − specificity) at various threshold settings, and the AUC summarizes this information into a single value between 0 and 1. A higher AUC indicates a better performance of the model in distinguishing between the classes.

In addition to the AUC, complementary metrics are employed to provide a more comprehensive understanding of the predictive performance. These include accuracy, precision, recall (sensitivity), specificity, F1-score, Matthews correlation coefficient (MCC), and the geometric mean (G-Mean). The notations and mathematical definitions of the metrics used in this study are summarized in Table 5.

### 3.7. Quantification of Heavy Metals Using Atomic Absorption Spectroscopy (AAS)

The metal concentration in the samples was determined using an atomic absorption spectrometer (Thermo Fisher Scientific iCE 3000 series, Waltham, MA, USA) operated with an air-acetylene flame, at 213.9 nm and 228.8 nm for Zn and Cd, respectively. The calibration curves were constructed employing an ID100 Autodilutor accessory of the Thermo Fisher Scientific company, (Waltham, MA, USA), which allows automatic sample dilution and working standards preparation for flame AAS. In this case, the working standards were prepared from a single master standard (50 mg/L for Zn, 100 mg/L for Cd). All samples were filtered through Whatman quantitative filter paper (2.5 µm) to retain microplastics and ensure the operating conditions of the atomic absorption equipment, which must guarantee the absence of particles. The seawater (SW) samples were additionally filtered with a membrane with a pore size of 0.45 µm. Once the calibration curve was constructed, the samples were measured using an ASX-280 Flame Autosampler manufactured by Teledyne CETAC Technologies, Omaha, NE, USA. The use of the autosampler enabled the simultaneous loading of the 23 samples by sequentially and uniformly performing the aspiration and rinse steps for each sample. Three measurements were recorded for both the working standards and the samples.

## 4. Experimental Results

The training processes were carried out on a system with a 13-generation Intel Core i9 CPU (Intel Corporation, Santa Clara, CA, USA), an NVIDIA GeForce RTX A2000 (Nvidia Corporation, Santa Clara, CA, USA) and 64 GB of RAM. Python 3.10 was used as the programming language, and Scikit-Learn served as the main library to implement the machine learning modules.

As previously defined, the models of Methods 1 and 3 were trained using 10% of the data, whereas for Method 2, only 0.1% was used due to its higher computational requirements caused by the significant increase in the number of inputs. Table 6 shows that, despite using only 0.1% of the data for Method 2, 11,000 entries with 7 predictors are generated. In contrast, Methods 1 and 3, which use 10% of the data, achieve 440 observations: 17,507 predictors for Method 1 and only 7 for Method 3. Furthermore, the training execution times demonstrate that Method 3 is more efficient in terms of computational resources, whereas Method 2 presents a notable disadvantage in this regard.

### 4.1. Method 1

**Figure 14:**
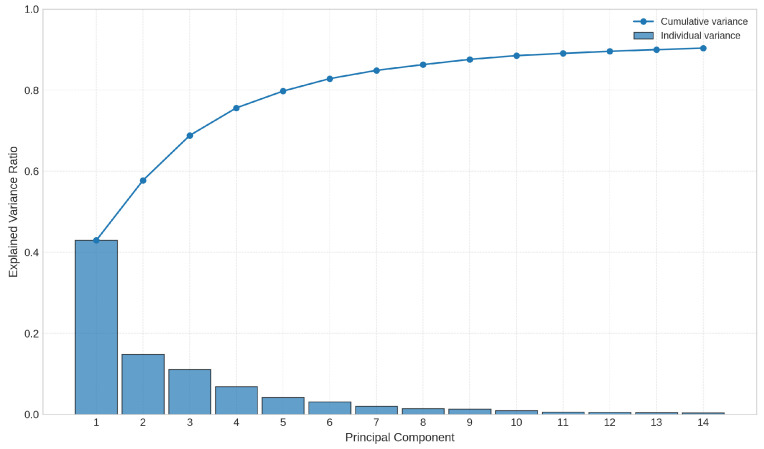
PCA scree plot showing both the individual and cumulative explained variance for the first principal components obtained using Method 1. Principal Component 1 (PC1) accounts for approximately 43% of the variance, while PC2 and PC3 explain about 15% and 11%, respectively. Together, these components capture a cumulative explained variance of approximately 69%.

A PCA analysis was performed, selecting the first three principal components for visualization. Figure 14 presents both the individual and cumulative explained variance. Principal Component 1 (PC1) accounts for approximately 43% of the variance, while PC2 and PC3 explain about 15% and 11%, respectively. Together, these three components capture a cumulative explained variance of approximately 69%.

**Figure 15:**
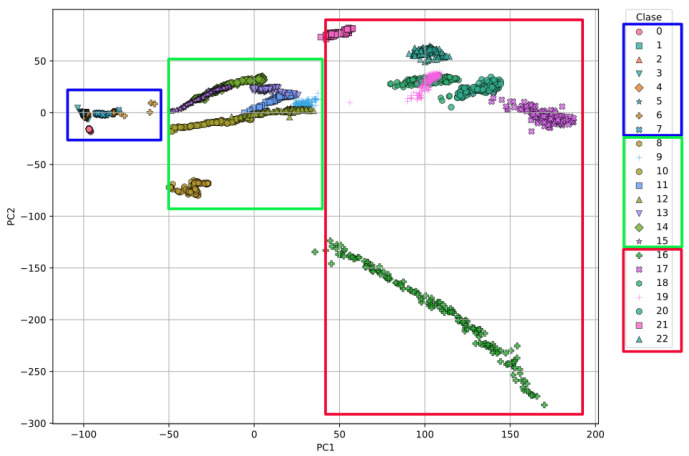
Visualization of the first principal components (PC1 vs. PC2) obtained through PCA applied to Deployment Method 1. Colored boxes highlight the grouping and separation of clusters by water type: blue for deionized water, green for laboratory-prepared saltwater, and red for seawater.

Figure 15 shows the projection of all individual measurements (replicates) in the space defined by the first two principal components (PC1 and PC2) obtained through Principal Component Analysis (PCA). Each class is therefore represented by multiple points, corresponding to repeated measurements, rather than a single aggregated value or centroid. The distribution highlights the relevance of the type of water in the clustering of the classes: in the blue box, all classes corresponding to deionized water are grouped; in the green box, those corresponding to synthetic water; and in the red box, those corresponding to seawater. This separation suggests that the variability captured by PC1 and PC2 is strongly correlated with the chemical composition of the water type.

**Figure 16:**
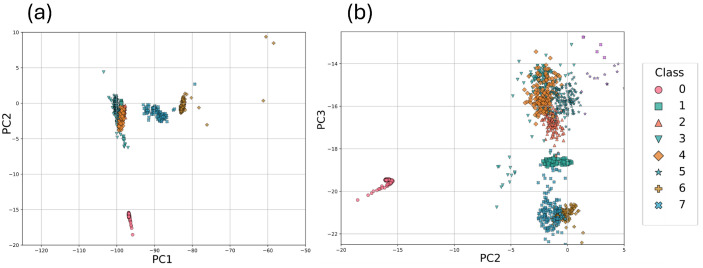
Projection onto (**a**) PC1-PC2 and (**b**) PC2-PC3 for the deionized water classes, which allows for a clearer view of the clustering and separation of the classes—a distinction that is not easily discernible in the previous figure due to the difference in scale. In both plots, there is a clear separation of class 0 (deionized water without contaminants) from the rest. In graph (**a**), class 1 appears to be lost among classes 2, 3, 4, and 5; however, by changing the perspective in graph (**b**), the separation of class 1 from the others becomes apparent. Classes 6 and 7 in graph (**a**) show a clear separation, whereas in (**b**) they appear to overlap.

In Figure 16a,b, the grouping of classes 0, 1, 2, 3, 4, 5, 6, and 7 corresponding to deionized water is more clearly observed in the PC1 vs. PC2 and PC2 vs. PC3 visualizations, respectively. In both cases, classes 0, 1, 6, and 7 exhibit a well-defined clustering separated from the rest, whereas classes 2, 3, 4, and 5 appear to overlap to some extent with each other.

**Figure 17:**
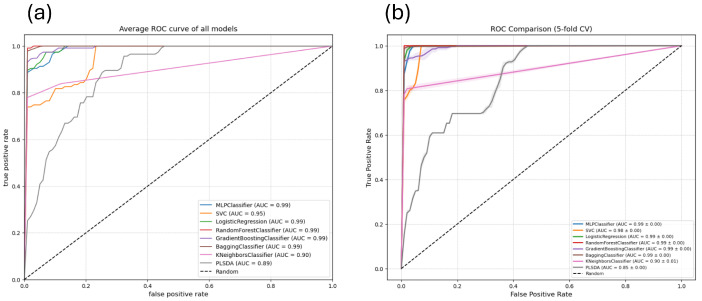
(**a**) Average ROC curves for eight classification models in method 1 using the test data set. Most models achieve AUC values close to 100%, except for the SVM model, which attains an AUC of 95%, as well as Keihbors and PLSDA, which achieved 90% and 89% accuracy, respectively for all individual curves). (**b**) ROC curve comparison with 5-fold cross-validation in method 1. By utilizing with train data set 5-fold cross-validation (5-fold CV), the curves include shaded bands representing the performance variance across different folds, offering a more robust estimation.

To evaluate the generalization ability and robustness of the proposed algorithms, their performance was analyzed through ROC curves using 5-fold cross-validation (5-fold CV) with the training dataset. Figure 17b shows performance curves along with their respective Area Under the Curve (AUC), incorporating shaded bands that represent the variance across folds to visualize the predictive stability of each model across different data partitions for all individual curves). Five models (MLP, Logistic Regression, Random Forest, Gradient Boosting, and Bagging) achieve an outstanding Area Under the Curve (AUC) of 0.99 ± 0.00, evidencing high stability and predictive capacity. The SVC model shows an improvement over simple evaluations, reaching an AUC of 0.98 ± 0.00. KNeighbors (0.90 ± 0.01) and PLSDA (0.85 ± 0.00) present the lowest performance.

Figure 17a shows the average ROC curve and the associated AUC value for each of the constructed models using the test data set. The graph reveals that five of the models, namely MLPClassifier, LogisticRegression, RandomForestClassifier, GradientBoostingClassifier, and BaggingClassifier, achieve an excellent and almost identical performance, with an AUC of 0.99. Their corresponding curves heavily overlap in the top-left corner of the plot, indicating a high discriminative capacity. The SVC follows the main group with an AUC of 0.95, followed by the KNeighborsClassifier with an AUC of 0.90, and PLSDA with an AUC of 0.89 for all individual curves).

**Figure 18:**
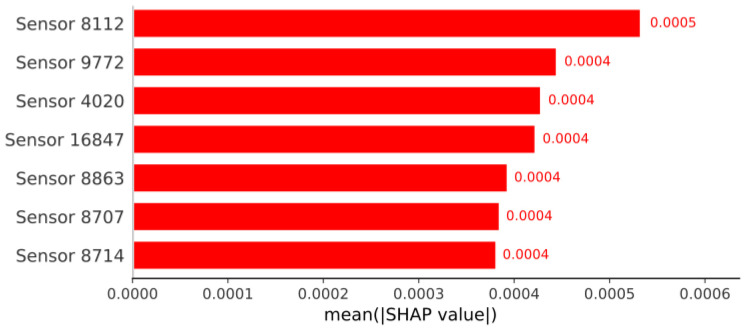
SHAP graphic of the Random Forest model for method 1.

The Figure 18 illustrates the importance of the features used by the Random Forest model, as evaluated using SHAP (SHapley Additive exPlanations) values, where each feature corresponds to a specific point within the signals generated by the seven sensors. Since each sensor contributes 2501 data points, which are organized sequentially from right to left from sensor 1 to sensor 7, it is possible to identify which sensor the most relevant features belong to. In this case, the SHAP analysis shows that among the most important features, those with IDs between 8000 and 10,000 predominate; these belong to sensor 4. In fact, the two most important features belong to this same sensor.

Table 7 presents the performance metrics of eight classification algorithms evaluated using “Method 1”. The results reveal that the ensemble models, specifically Random Forest and Bagging, exhibit the most outstanding performance, both achieving an accuracy of 0.991 and exceeding 0.99 in the majority of their metrics. Algorithms such as Gradient Boosting, Logistic Regression, SVM, and MLP Classifier are positioned at an intermediate to high level, with accuracies ranging between 0.844 and 0.913. In contrast, the PLSDA model shows a notably poor performance compared to the rest, recording the lowest accuracy in the comparison with only 0.260.

### 4.2. Method 2

In this method, the first three principal components from PCA capture a cumulative explained variance of approximately 81%, where PC1 accounts for about 52% and PC2 for approximately 18%.

**Figure 19:**
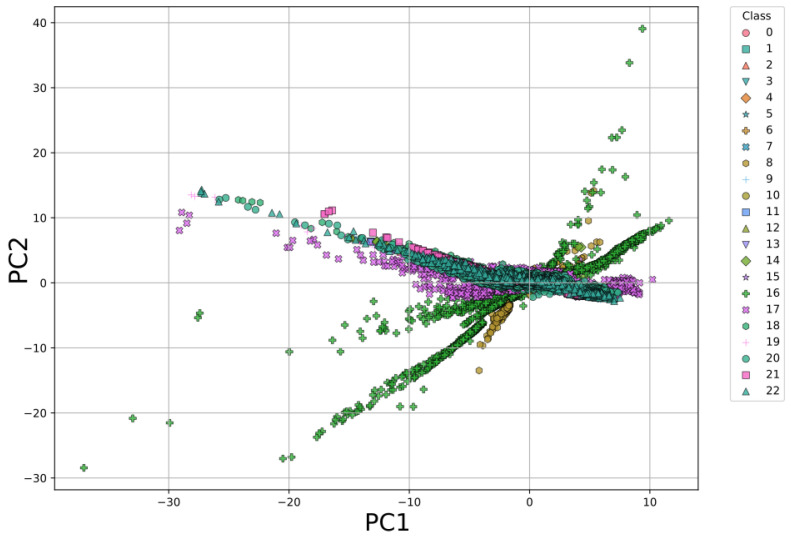
PCA projection (PC1 vs PC2) for Method 2, showing overlap of classes, particularly those corresponding to seawater, and the convergence of all classes toward a single point in the plane.

The visualization of the data in the space defined by the principal components PC1 and PC2, obtained through PCA applied with Method 2, is shown in Figure 19. In this case, the data for all classes are clustered together, overlapping in the same space, which prevents a clear separation between them from being observed.

**Figure 20:**
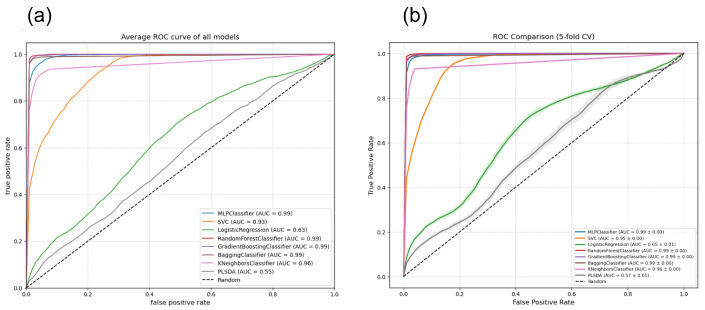
(**a**) Average ROC curves for eight classification models (Method 2). The plot shows the diagnostic performance of each algorithm. (**b**) Average ROC curves with 5-fold cross-validation (Method 2) with the train data set. The plot illustrates the true positive rate versus the false positive rate for eight classifiers, including the fold variance bands.

Figure 20b presents the comparative evaluation of the ROC curves using 5-fold cross-validation using the train data set in method 2 for all individual curves). This configuration reveals a significant drop in the performance of certain linear models compared to method 1, specifically in Logistic Regression (AUC = 0.65 ± 0.01) and PLSDA (AUC = 0.57 ± 0.01), whose curves approach the random classification threshold. In contrast, ensemble and non-linear models, such as Random Forest, Bagging, Gradient Boosting, and MLP Classifier, demonstrate remarkable robustness against these variations, maintaining an outstanding performance with an AUC of 0.99 ± 0.00.

Figure 20a illustrates the inefficiency of logistic regression and PLSDA for the second method in terms of AUC, yielding values close to the random classifier for all individual curves). The Random Forest, Gradient Boosting, Bagging, and MLP Classifier models present the highest discriminative power (AUC = 0.99), followed by KNeighbors (AUC = 0.96) and SVC (AUC = 0.93). Conversely, Logistic Regression (AUC = 0.63) and PLSDA (AUC = 0.55) show a considerably lower performance.

Table 8 details the performance metrics of the eight classification algorithms evaluated under “Method 2”. The results reveal that the Random Forest model leads the comparison, achieving the highest performance with an accuracy of 0.948 and an AUC of 0.999. It is closely followed by other robust algorithms such as Gradient Boosting, Bagging, and MLP Classifier, which maintain an accuracy above 0.866 and AUC values over 0.99. In sharp contrast, linear-based models experience a severe degradation under this configuration. Logistic Regression and SVC drastically drop to accuracies of 0.145 and 0.485 respectively, while the PLSDA model exhibits the worst behavior in the table, with a near-zero accuracy of 0.061 and F1-score and MCC metrics close to zero.

**Figure 21:**
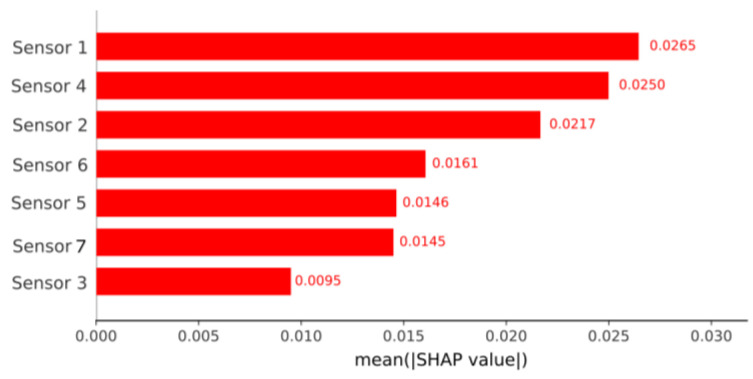
Global feature importance (SHAP values) for the Random Forest model (Method 2). The bar chart illustrates the mean absolute contribution of each sensor to the model’s predictions. Sensor 1 and Sensor 4 exhibit the highest impact on the system’s discriminative capacity, followed by Sensor 2. Conversely, Sensor 3 presents the lowest overall influence on the algorithm’s classification decisions.

To transparently interpret the decisions of the best-performing model under Method 2 (Random Forest), the SHAP explainability method was employed. Figure 21 presents the global feature importance, calculated from the mean absolute SHAP value for each sensor. This analysis allows for the direct identification of which variables exert the greatest influence on the algorithm’s predictions, revealing that sensors 1 and 4 are the most determinant for the classification process.

### 4.3. Method 3

The three principal components selected from PCA in Method 3 capture a cumulative explained variance of approximately 89%. The first two components together explain about 81% of the variance, with PC1 accounting for approximately 58% and PC2 about 23%.

**Figure 22:**
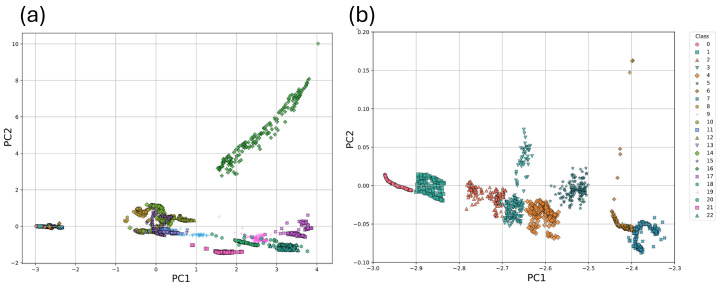
(**a**) Projection of the samples in the space defined by PC1 and PC2, obtained through PCA applied to the full dataset of Method 3. (**b**) PC1 vs. PC2 projection for Method 3 with a zoomed-in view of the region corresponding to the water classes.

Figure 22 shows the clustering of each class in the PC1 vs. PC2 components of the performed PCA. A clear separation is observed for each class.

**Figure 23:**
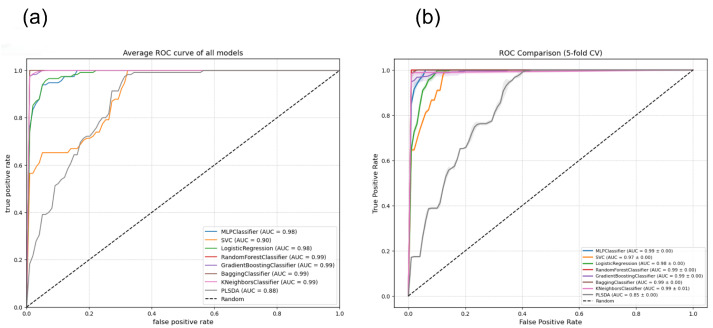
(**a**) ROC curves for the classification models evaluated in Method 3 using the test data set. The true positive rate (TPR) is plotted against the false positive rate (FPR). (**b**) ROC curve comparison with 5-fold cross-validation (Method 3) with the train data set. The plot illustrates the classification performance of the eight models under the third experimental configuration, including the cross-fold variance bands.

To evaluate the impact of method 3 on the predictive capacity of the algorithms, ROC curves were generated using 5-fold cross-validation in the training set. Figure 23b demonstrates that this new configuration stabilizes and maximizes overall performance for all individual curves). The majority of the classifiers (Random Forest, MLP, Gradient Boosting, Bagging, KNeighbors, and Logistic Regression) achieve an exceptional Area Under the Curve (AUC) between 0.98 and 0.99. The SVC model maintains high performance (AUC = 0.97), while PLSDA (AUC = 0.85) presents the lowest level in the comparison, although it evidences a significant recovery compared to previous methods.

Figure 23a shows the high performance of the seven models evaluated using unfolding Method 3, with outstanding AUC values in all cases, even after the reduction of predictors. The PLS-DA model obtained the lowest value, with an AUC of 88% for all individual curves).

Table 9 presents the detailed performance metrics for the eight classification algorithms evaluated under Method 3. The results quantitatively confirm the superiority of the Random Forest model, which achieves optimal performance with perfect values of 1.000 across all evaluated metrics, including accuracy, precision, F1-score, and AUC. Similarly, the Bagging model demonstrates exceptional results with a perfect AUC of 1.000 and an accuracy of 0.991. It is important to highlight that linear models such as Logistic Regression and SVC show solid performance under this configuration, achieving accuracies of 0.765 and 0.774, respectively, accompanied by outstanding AUC values (0.987 and 0.907). Conversely, the PLSDA model remains the lowest-performing algorithm in the comparison, recording an accuracy of only 0.174 and an F1-score of 0.062.

Overall, the results suggest that the ensemble models provide more robust and balanced performance for this classification task.

**Figure 24:**
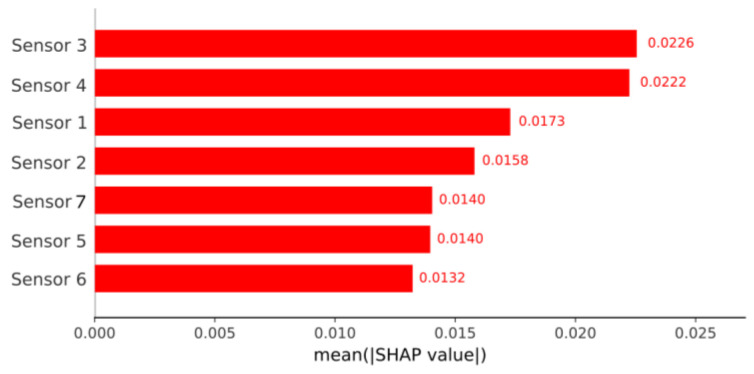
Global feature importance (SHAP values) for the Random Forest model (Method 3). In this evaluation, Sensor 3 and Sensor 4 register the highest impact on the discriminative capacity of the model, followed by Sensor 1.

To understand the internal dynamics of the decisions made by the Random Forest model under the Method 3 configuration, a global interpretability analysis was conducted using SHAP values. Figure 24 details the relative importance of each feature in the classification process. It is notable to observe a significant shift in the variable hierarchy compared to previous configurations: under this processing strategy, Sensor 3 emerges as the most determinant factor for the algorithm’s predictions, revealing a new dependency on the data patterns.

### 4.4. Heavy Metals Analysis Using Atomic Absorption Spectroscopy (AAS)

**Figure 25:**
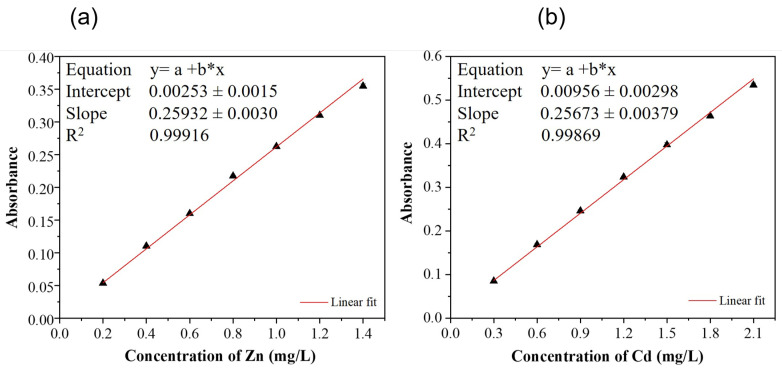
Calibration curve for (**a**) Zn and (**b**) Cd by AAS.

Figure 25 shows the calibration curves, the linear regression equations, and the correlation coefficients (R^2^) for working standard solutions of zinc (Figure 16a) and Cd (Figure 16b). The calibration curves showed a strong linear relationship between concentrations and absorbance, with correlation coefficients of 0.99916 for Zn and 0.99869 for Cd. The limits of detection (LOD) and limits of quantitation (LOQ) were defined as thrice and ten times the standard deviation of the blank, respectively [66]. Table 9 presents the metal concentrations calculated from the linear equations, where <LOD is indicated when the absorbance reading was below the limit of detection. In this case, the solution does not contain the analyte (Zn or Cd), or its concentration is too low for the instrument to measure reliably.

The adsorption capacity of microplastics (*q*) was calculated using Equation (2) given below [67]:

(5) $$q=\frac{V(C_{in}-C_{t})}{m}$$

where *q* (μg of metal/g of MP) is the adsorption capacity of heavy metals; V(L) is the volume of heavy metal solution; m(g) is the mass of MP; $C_{in}$ in (μg/L) and $C_{t}$ (μg/L) are the concentrations of the solutions before and after contact with the MP.

The results in Table 10 show that the capacity of expanded polystyrene (ESP) microplastic to adsorb Cd and Zn ions varies according to the characteristics of the matrix (type of water). For the samples in deionized water, the microplastics showed greater adsorption ability to Cd than to Zn, even when the two metals were simultaneously dissolved in the matrix, with *q* values of 82.4 µg Zn/g MP and 421.1 µg Cd/g MP. Furthermore, the Cd adsorption capacity of MPs in synthetic saltwater (*q* = 3525.1 µg Cd/g MP) exceeded that in seawater (*q* = 211.6 µg Cd/g MP). This could be explained by salinity-driven aggregation of MP and competitive adsorption of multiple ions present in seawater (Na^+^, Cl^−^, Mg^2+^, SO_4_^2−^, Ca^2+^, K^+^, HCO_3_^−^, Sr^2+^, among others) as evidenced by higher electrical conductivity values (47.51 ms/cm for SW + Cd + Zn + MP and 13.19 ms/cm for SSW + Cd + Zn + MP) [68]. Other authors, for example, Chen, P.W., et al. (2024), have reported the adsorption capacity of microplastics using indirect experiments based on measuring metal concentrations in solutions after contact with microplastics [67]. In various studies, the application of different analytical techniques such as scanning electron microscopy (SEM) and X-ray photoelectron spectroscopy (XPS) has demonstrated that metals, including Pb, Cu, Cr, Cd, Ni, Al, Co, Zn, Mn, Fe, Ca, Ag, and Hg, adhere to the surface of microplastics (MPs). This adsorption process is governed by physical, chemical, and indirect mechanisms, influenced by the type of polymer, metal properties, and environmental conditions. Physical adsorption occurs through van der Waals forces, electrostatic interactions, and pore filling, and is enhanced by surface roughness and area, particularly after aging. Chemical adsorption involves complexation with oxygen-containing functional groups (–COOH, –OH), as well as ion exchange and the formation of more stable bonds. Indirect adsorption, on the other hand, is mediated by biofilms on the surface, whose extracellular polymeric substances provide additional active sites. Collectively, these mechanisms enable microplastics to act as vectors for metals in the environment [69,70].

## 5. Discussion

A consistent pattern across all three configurations is the exceptional robustness of tree-based and ensemble models (Random Forest, Gradient Boosting, Bagging), as well as neural networks (MLP). These algorithms managed to maintain an Area Under the Curve (AUC) above 0.98 in nearly all evaluated scenarios, regardless of the method used.

In contrast, linear models (Logistic Regression, PLSDA) showed a high degree of sensitivity to the data processing strategy. While they performed highly competitively in the initial configuration, the application of “Method 2” caused a severe collapse in their discriminative capacity, reducing the accuracy of Logistic Regression to 0.145 and that of PLSDA to a near-zero margin of 0.061. This decline suggests that Method 2 introduces a transformation in the data that destroys the linear separability of the classes, an obstacle that nonlinear algorithms (such as Random Forest) manage to overcome without difficulty.

**Figure 26:**
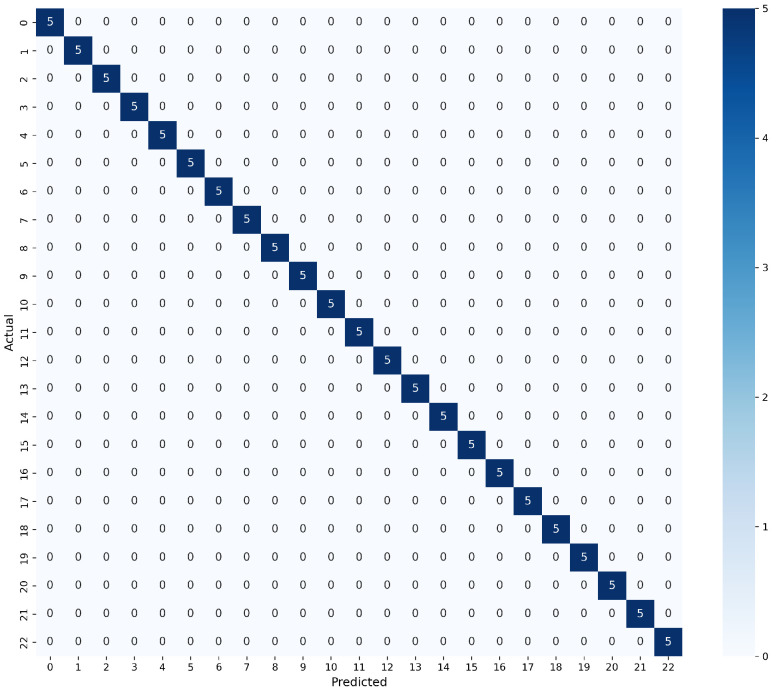
Confusion matrix RF method 3.

The implementation of Method 3 proved to be the most effective and comprehensive strategy. Not only did it restore the predictive power of the linear models (raising the AUC of the logistic regression back to 0.98), but it also maximized the performance of the more robust models. Under this paradigm, the Random Forest model achieved perfect classification (1.000 in accuracy, precision, recall, and AUC). This indicates that Method 3 projects the features into a space where the decision boundaries are simultaneously traceable by simple hyperplanes (favoring linear models) and highly definable by conditional splits (favoring decision trees). Figure 26 shows the confusion matrix resulting from the tests on the RF model (for the matrices corresponding to all models in each method.

Although Method 3 successfully improved the signal-to-noise ratio and highlighted the most relevant sensor features, the Partial Least Squares Discriminant Analysis (PLS-DA) model failed to achieve a competitive performance compared to non-linear algorithms like Random Forest. This persistent underperformance is primarily driven by the inherent limitations of linear models when navigating high-dimensional, multiclass feature spaces. Fundamentally, PLS-DA assumes linear decision boundaries, whereas the underlying relationship between the sensor responses and the 22 target classes is highly non-linear, and a complex topology where tree-based ensembles naturally excel. Furthermore, when addressing 22 simultaneous categories, PLS-DA is severely compromised by the “masking effect,” a geometric deficit where classes located in the middle of the feature space are eclipsed by extreme classes, which no degree of preprocessing can fully resolve.

Based on the results obtained, it was found that the performance of the models created using method 1 generally presents better minimum and maximum values for each metric, as can be seen by comparing Table 7, Table 8 and Table 9, presenting similar values in accuracy and AUC, but standing out in precision, recall, MCC, and geometric mean, compared to the other methods.

The best performance was obtained by the models generated with method 1, with the RF model standing out, which showed scores of 0.94 and above in all the evaluated metrics. Figure 26 shows the confusion matrix resulting from the tests on this model. It is noticeable how it only makes erroneous predictions in classes 2, 3, and 4, corresponding to pure water with Cd, pure water with Cd plus microplastics, and pure water with Zn, respectively. For class 2, it makes 2 out of 5 erroneous predictions, both of which are related to class 4. For class 3, there are also two erroneous predictions, one related to class 1 (pure water with microplastics) and the other to class 5 (pure water with Zn and microplastics). While for class 4 there is only one confusion, and it is with class 3. When analyzing the conductivity measured for these classes (see Table 10), values on the order of micro siemens are observed with values close to 4.58–10.68, which could explain the difficulty of correct classification.

On the other hand, Method 2 showed the lowest performance, obtaining the lowest values (see Table 8). The SVM and Logistic Regression models, in particular, had the greatest difficulty predicting classes positively, as their accuracy, recall, and F1 score were all better than 0.5. This poor performance is corroborated by comparing metrics that integrate the ability to identify positive and negative classes. The geometric mean for these two models was only 0.03 and 0.28, respectively, indicating that they can correctly classify some classes but poorly classify others. Analyzing only this metric, it is observed that in Method 1, SVM again presented the lowest value, 0.16, and in Method 3, 0.42 (see Table 9), followed very closely by Logistic Regression with 0.41, thus confirming that these are the models with the lowest performance.

In terms of computational cost, method 2 presents significant disadvantages compared to methods 1 and 3, since, in general, it requires greater computational capacity but yields significantly lower results, as mentioned above.

The models generated using Method 3 generally performed well across all metrics, despite the drastic reduction in predictors compared to Method 1. However, a comparison reveals a significant reduction in the performance of the MLP Classifier and Logistic Regression models, particularly in precision, recall, F1 score, MCC, and geometric mean. Each of these models moved from values of 0.9 or higher in these metrics in Method 1 to values below 0.64 and 0.42 in Method 3. SVM, while showing a slight improvement, continued to exhibit appreciably poor values, remaining below 0.5 in the metrics, except for accuracy (0.97) and AUC (0.91). See Table 7 and Table 9.

A very important point to highlight is the clear separation of the different types of water in the PCA-2D visualization in Figure 15. In this figure, it is possible to observe how a small grouping is generated for the classes corresponding to pure water, a medium one for those corresponding to synthetic salt water, and a last, much larger one for the classes corresponding to seawater. This behavior could be because the three groups of water samples are well-differentiated in their physicochemical properties, both in terms of electrical conductivity (EC), which is the ability of water to conduct an electrical current (related to the concentration of ions in a solution), and pH, which is a measure of the acidity or alkalinity (basicity) of an aqueous solution. According to Table 8, there are significant differences in electrical conductivity (EC) among the classes: deionized water, synthetic salt water, and seawater. The DW samples show low electrical conductivity values (around 4.58–503.4 µs/cm), while the samples from the SSW and SW groups exhibit higher EC values close to 13 ms/cm and 47 ms/cm, respectively.

Additionally, previous research recognizes that the adsorption behavior of heavy metals onto microplastics is affected by various factors such as the type of microplastics, particle size, pH, temperature, and salinity (EC is a measure of salinity) [71,72]. This study showed that the adsorption capacity of microplastics was influenced by the physicochemical properties of heavy metals (Zn or Cd) and by the presence of salts, dissolved organic substances, and other metals, which alter the water’s physicochemical properties.

The results showed that a basic medium with a pH of 8.24–9.39 (for SSW samples) enabled greater metal adsorption capacity on microplastics. According to the literature, different Cd species can exist within this pH range, including Cd^2+^ in greater proportion (∼80%), in addition to CdOH^+^ and Cd(OH)_2_ in a smaller proportion. Therefore, the presence of these hydrolyzed species and the decrease in the amount of hydrogen ions modify the electrostatic interactions between the metal and the microplastic surface, favoring its adsorption capacity. Whereas under highly acidic conditions (pH ≈ 2), there are predominantly free divalent cations (Cd^2+^); therefore, less removal is expected, which can be attributed to the increased competition between hydrogen ions and metal cations in solution. On the other hand, the zinc chemical speciation indicates that above 7, the fraction of Zn^2+^ in aqueous solution decreases (almost 20% at pH 9) and starts the formation of Zn_2_(OH)^3+^, ZnOH^+^ and Zn(OH)_2_. These results are consistent with previous reports in the literature, which have shown that pH variations influence metal adsorption behavior, because competition between ions H^+^ and metals for adsorption sites on the surface of MPs occurs. Therefore, this situation will be differentiated by having a lower or higher concentration of these H^+^ ions in solution [73,74]. More studies are needed to understand the phenomena of adsorption in real samples, especially in marine waters, which exhibit complex characteristics due to the presence of multiple components, such as minerals, organic matter and sediments.

## 6. Conclusions

This study successfully developed a machine learning framework for classifying deionized water, synthetic, and seawater samples contaminated with microplastics and heavy metals using an electronic tongue system. Through systematic comparison of state-of-the-art classification models across three data preprocessing methods and corroboration with chemical analyses, we demonstrated that machine learning can substantially improve the accuracy of contaminated water classification.

The models used in this work capture global response patterns rather than direct concentration measurements, which may partially explain the matrix-related effects. The proposed system is not intended to estimate total metal concentrations, but rather to classify samples based on their compositional differences. The successful discrimination between water and water containing microplastics confirms that MPs constitute a significant factor that influences the electrochemical response. Therefore, classification performance is based on the overall composition of the system, including possible interactions between contaminants, rather than on absolute concentration values.

Our comparative analysis revealed that method 1 yielded superior classification performance with metric values exceeding 0.85, while also demonstrating greater computational efficiency compared to methods 2 and 3. Method 2 proved computationally prohibitive, requiring weeks for SVM execution due to its complex data decomposition structure, and consistently produced the lowest performance metrics. The consistently poor performance of Logistic Regression across all methods indicates that the classification problem is inherently non-linearly separable, requiring more sophisticated decision boundaries than linear models can provide.

Random Forest emerged as the most robust and stable classification model, consistently ranking among the top performers across all three preprocessing methods and achieving or closely approaching optimal results in most cases. This confirms its effectiveness for classification tasks requiring a balance between precision, recall, and model complexity.

Then, the concentrations of Zn and Cd determined by atomic absorption spectroscopy support the results from machine learning algorithms for detecting and classifying water samples containing both microplastics and heavy metals. Likewise, MPs exhibit a strong adsorption capacity of Zn and Cd onto MPs, which was observed in all three types of water studied. However, the accumulation of heavy metals on MPs in real aquatic environments requires further study, as adsorption mechanisms are influenced by both the physical and chemical characteristics of MPs and the conditions of the surrounding water (pH, electrical conductivity, metal concentration, presence of organic matter, etc.).

Although this work demonstrates that it is possible to detect these polluting agents and that the methodology yields good results, in the future, further studies are expected to be carried out to define the impact on the number of sensors, their distribution, reference electrode material, concentrations of microplastics and heavy metals, among other factors.

Finally, it is worth noting that, given this work’s focus on a pattern recognition perspective, we did not conduct a sensor selection study or a final analysis to determine which sensor is more sensitive to microplastics or heavy metals. The idea was to determine whether, under the same conditions and with a defined number and type of sensors, it is possible to detect microplastics and heavy metals in the samples. Results show that although not all sensors are made of different materials, it is still possible to achieve good identification. As future work, a sensitivity analysis of the sensors can be applied to continue improving the results.

## Figures and Tables

**Table 1:** Working electrode material of the seven (7) sensors used in the electronic tongue.

Sensor	Working Electrode Material
1	Gold
2	Platinum
3	Graphite
4	Silver
5	Gold-Platinum Alloy
6	Gold
7	Gold

**Table 2:** Combination of samples analyzed. Deionized water (DW), synthetic saltwater (SSW), seawater (SW), Cd (1.5 ppm), Zn (1 ppm), microplastic expanded polystyrene (MP).

Base Sample	Combination	Measurements	Class ID
Deionized Water (DW)	DW	200	0
	DW + MP	200	1
	DW + Cd	200	2
	DW + Cd + MP	200	3
	DW + Zn	200	4
	DW + Zn + MP	200	5
	DW + Cd + Zn	200	6
	DW + Cd + Zn + MP	200	7
Synthetic Saltwater (SSW)	SSW	200	8
	SSW + MP	200	9
	SSW + Cd	200	10
	SSW + Cd + MP	200	11
	SSW + Zn	200	12
	SSW + Zn + MP	200	13
	SSW + Cd + Zn	200	14
	SSW + Cd + Zn + MP	200	15
Seawater (SW)	SW	200	16
	SW + MP	200	17
	SW + Cd	200	18
	SW + Cd + MP	200	19
	SW + Zn	200	20
	SW + Zn + MP	200	21
	SW + Cd + Zn + Mp	200	22

**Table 3:** Dataset construction and validation split by preprocessing method. Original dataset: 23 classes × 200 measurements × 7 sensors × 2501 sample points. Cross-validation: 5-fold, applied over the training subset only.

Method	Original Samples	Data Used (%)	Samples Used	Training (80%)	Test (20%)	Predictors	CV Folds
Method 1	4600	10%	460	368	92	17,507 (J × K)	5
Method 2	4600	0.1%	46	11,040 ^†^	2760 ^†^	7 (*J*)	5
Method 3	4600	10%	460	368	92	7 ($J_{\max}$)	5

J = 7 sensors; K = 2501 sample points per sensor; I = 200 measurements per class; Q = 23 classes. Original samples =Q × I = 23 × 200 = 4600. ^†^ Method 2 expands rows by *K*: each measurement unfolds into *K* rows, resulting in a significantly larger row count despite fewer input samples.

**Table 4:** Hyperparameter search space for each classification model. Optimization was performed via grid search with a 5-fold cross-validation.

Model	Hyperparameter	Values/Range
MLP	hidden_layer_sizes	(50,50,50), (50,100,50), (10,)
	activation	relu, tanh
	solver	sgd, ada
	alpha	0.0001, 0.05
	learning rate	constant, adaptive
SVM	C	1, 10, 100, 1000
	kernel	rbf, linear
	gamma	scale, 1, 0.1, 0.001, 0.0001
	class weight	balanced, none
Logistic Regression (baseline model)	C	0.01, 0.1, 1, 10, 100
	solver	lbfgs, liblinear
	class weight	balanced, none
Random Forest	n_estimators	10, 50, 100, 200
	max_depth	10, 20, 30, None
	class weight	balanced, none
	min_samples_split	2, 5, 10
Gradient Boosting	n_estimators	10, 50, 100, 200
	learning_rate	0.01, 0.1, 0.2
	max_depth	3, 5, 10
	min_samples_split	2, 5, 10
Bagging	n_estimators	10, 50, 100, 200
	max_samples	0.5, 0.7, 1.0
	max_features	0.5, 0.7, 1.0
K-Nearest Neighbors	n_neighbors	3, 5, 7, 10
	weights	uniform, distance
	metric	euclidean, manhattan

**Table 5:** Notations and descriptions of performance metrics.

Notation	Description	Notation	Description
TP	True positives	Accuracy	$\frac{TP\;+\;TN}{TP\;+\;TN\;+\;FP\;+\;FN}$
FP	False positives	Precision	$\frac{TP}{TP\;+\;FP}$
TN	True negatives	Specificity	$\frac{TN}{TN\;+\;FP}$
FN	False negatives	MCC	$\frac{TP\;\cdot \;TN\;-\;FP\;\cdot \;FN}{\sqrt{\left(TP\;+\;FP)(TP\;+\;FN)(TN\;+\;FP)(TN\;+\;FN\right)}}$
Recall	$\frac{TP}{TP\;+\;FN}$	G-Mean	$\sqrt{\mathrm{Sensitivity}\;\cdot \;\mathrm{Specificity}}$
F1 Score	$2\;\cdot \;\frac{\mathrm{Precision}\;\cdot \;\mathrm{Recall}}{\mathrm{Precision}\;+\;\mathrm{Recall}}$	AUC	Area under ROC curve

**Table 6:** Comparative analysis of the methods with respect to data proportion used, number of predictors, number of entries, and execution time.

Method	Dataset Proportion	Nro. de Predictors	Nro. of Inputs	Execution Time
Method 1	10%	17,507	440	aprox. 3 min
Method 2	0.1%	7	11,000	aprox. 6 min
Method 3	10%	7	440	aprox. 1 min

**Table 7:** Performance metrics of classification algorithms using Method 1.

Model	Accuracy	Precision Avg.	Recall Avg.	F1 Score Avg.	AUC Avg.	MCC Avg.	Geometric Mean Avg.
MLP Classifier	0.844	0.853	0.844	0.830	0.991	0.831	0.865
SVM	0.844	0.860	0.844	0.840	0.960	0.840	0.883
Logistic Regression	0.887	0.855	0.887	0.868	0.993	0.865	0.907
Random Forest	**0.991**	**0.993**	**0.991**	**0.991**	**0.999763**	**0.991**	**0.995**
Gradient Boosting	0.913	0.927	0.913	0.914	0.994	0.913	0.949
Bagging	0.991	0.992	0.991	0.991	0.999289	0.991	0.995
K. Neighbors	0.791	0.772	0.791	0.756	0.906	0.764	0.797
PLSDA	0.260	0.110	0.261	0.14	0.887	0.151	0.243

**Table 8:** Performance metrics of classification algorithms using Method 2.

Model	Accuracy	Precision Avg.	Recall Avg.	F1 Score Avg.	AUC Avg.	MCC Avg.	Geometric Mean Avg.
MLP Classifier	0.866	0.868	0.866	0.865	0.994	0.860	0.925
SVC	0.485	0.520	0.485	0.479	0.931	0.470	0.642
Logistic Regression	0.145	0.163	0.145	0.129	0.631	0.104	0.313
Random Forest	**0.948**	**0.949**	**0.948**	**0.948**	**0.999**	**0.946**	**0.972**
Gradient Boosting	0.931	0.932	0.931	0.931	0.998	0.928	0.963
Bagging	0.929	0.929	0.929	0.928	0.993	0.925	0.962
K. Neighbors	0.766	0.771	0.766	0.764	0.960	0.756	0.864
PLSDA	0.061	0.015	0.061	0.020	0.555	0.008	0.097

**Table 9:** Performance metrics of classification algorithms using Method 3.

Model	Accuracy	Precision Avg.	Recall Avg.	F1 Score Avg.	AUC Avg.	MCC Avg.	Geometric Mean Avg.
MLP Classifier	0.852	0.831	0.852	0.822	0.986	0.828	0.875
SVC	0.774	0.792	0.774	0.746	0.907	0.755	0.822
Logistic Regression	0.765	0.735	0.765	0.732	0.987	0.733	0.797
Random Forest	**1.000**	**1.000**	**1.000**	**1.000**	**1.000**	**1.000**	**1.000**
Gradient Boosting	0.939	0.959	0.939	0.939	0.999	0.942	0.965
Bagging	0.991	0.993	0.991	0.991	**1.000**	0.991	0.995
K. Neighbors	0.930	0.936	0.930	0.929	0.999	0.928	0.960
PLSDA	0.174	0.039	0.174	0.062	0.875	0.071	0.155

**Table 10:** Heavy metals concentration.

Class ID	Combination (Sample)	Measured Metal	Mean Absorbance	RSD (%)	Metal Concentration (mg/L)	*q* (μg Metal/g MP)	pH	Conductivity
1	DW + MP	Zn	0.00172	-	<LOD	-	3.77	4.58 μs/cm
		Cd	0.00037	-	<LOD	-		
3	DW + Cd + MP	Zn	0.00319	-	<LOD	-	3.22	10.68 μs/cm
		Cd	0.35300	0.80	1.33776	403.2		
5	DW + Zn + MP	Zn	0.24654	0.39	0.94094	132.3	3.43	197.3 μs/cm
		Cd	0.00072	-	<LOD	-		
7	DW + Cd + Zn + MP	Zn	0.25325	0.89	0.96684	82.4	2.89	503.4 μs/cm
		Cd	0.35115	0.56	1.33054	421.1		
9	SSW + MP	Zn	0.00307	-	<LOD	-	9.39	13.2 ms/cm
		Cd	0.00061	-	<LOD	-		
11	SSW + Cd + MP	Zn	0.00331	-	<LOD	-	8.24	13.28 ms/cm
		Cd	0.00337	1.18	0.09197	3493.9		
13	SSW + Zn + MP	Zn	0.00704	0.40	0.38150	1523.2	8.85	13.15 ms/cm
		Cd	0.00011	-	<LOD	-		
15	SSW + Cd + Zn + MP	Zn	0.02994	0.17	0.65053	868.0	8.30	13.19 ms/cm
		Cd	0.00299	1.18	0.07940	3525.1		
17	SW + MP	Zn	1.09000	0.45	0.40876	-	2.63	47.46 ms/cm
		Cd	0.00046	-	<LOD	-		
21	SW + Zn + MP	Zn	0.31867	0.99	1.23016	483.5	2.43	47.59 ms/cm
		Cd	0.00022	-	<LOD	-		
22	SW + Cd + Zn + MP	Zn	0.05454	0.75	0.32600	2697.7	2.39	47.51 ms/cm
		Cd	0.37288	0.21	1.41519	211.6		

RSD: Relative Standard Deviation. *q*: Adsorption capacity of heavy metals (μg metal/g MP). LOD (Zn) = 0.00366 mg/L, LOQ (Zn) = 0.01222 mg/L. LOD (Cd) = 0.00187 mg/L, LOQ (Cd) = 0.00622 mg/L.

## Data Availability

Dataset available on request from the authors.

## Abbreviations

The following abbreviations are used in this manuscript:

AAS	Atomic Absorption Spectroscopy
AUC	Area Under the Curve
AUC-ROC	Area Under the Curve–Receiver Operating Characteristic
DW	Deionized Water
EPS	Expanded Polystyrene
G-mean	Geometric Mean
HM	Heavy Metals
ICP-MS	nductively Coupled Plasma Mass Spectrometry
ICP-OES	Inductively Coupled Plasma Optical Emission Spectroscopy
KNN	K-Nearest Neighbors
LOD	Limit of Detection
LOQ	Limit of Quantification
MCC	Matthews Correlation Coefficient
ML	Machine Learning
MLP	Multilayer Perceptron
MP	Microplastics
NAA	Neutron Activation Analysis
PCA	Principal Component Analysis
PET	Polyethylene Terephthalate
RF	Random Forest
SSW	Synthetic Seawater
SVM	Support Vector Machine
SW	Seawater
XRF	X-ray Fluorescence
